# Immune monitoring technology primer

**DOI:** 10.1186/s40425-015-0086-9

**Published:** 2015-10-20

**Authors:** Kevin K. Dobbin

**Affiliations:** College of Public Health, University of Georgia, Athens, GA 30602 USA

## Abstract

**Background:**

Recent biotechnological developments have resulted in increasing interest in immunology biomarkers. These biomarkers have potential clinical utility in the near future as predictors of treatment response. Hence, clinical validation of these predictive markers is critical.

**Findings:**

The process of clinically validating a predictive biomarker is reviewed. Validation of a predictive biomarker requires quantifying the strength of a statistical interaction between marker and a treatment. Different study designs are considered.

**Conclusions:**

Clinical validation of immunology biomarkers can be demanding both in terms of time and resources, and careful planning and study design are critical.

## Findings

### Description of the technology

Appropriate statistical design and data of experiments is necessary for the successful development of predictive immunology biomarkers. Predictive immune response-based biomarkers may provide “evidence about the probability of benefit or toxicity” [1] from an immunotherapy approach. This discussion will focus on biomarkers for benefit. An ideal predictive marker would allow us to perfectly separate patients into a group who will respond to the therapy, and a group who will not respond. The groups are formed based on biomarker measurements taken either before any therapy is given, or early on in the treatment regimen.

Predictive markers in the real world do not attain the ideal sketched in the previous paragraph, and clinical validation is the process to determine how these imperfect markers, if actually used in the clinic, will impact clinical outcomes. In general, this is a complicated question and it is helpful to break it down into simpler parts. *First*, the test needs to be analytically validated. Here, much guidance has been provided in recent years for assessing and reporting technical reproducibility [2, 3]. Although immunology markers present unique technical measurement challenges, the guidance in these papers may be helpful. *Second*, the analytically validated test needs to be clinically validated. For a predictive biomarker validation study in a phase III setting, a clinically relevant outcome should be used, one that reflects “survival or symptomatic status of the subject” [4], or an approved surrogate outcome that has been adequately validated. Few such surrogates exist, however. New end points need to be defined that capture immunotherapy related response patterns such as delayed responses, “progression before regression and delayed survival separation curves.” *Third*, an appropriate study design needs to be put in place and analyzed to validate the predictive marker. Such a study may be either prospective or use archived specimens from a clinical trial [5]. How to properly design and analyze a biomarker validation study is currently an area of active development, which we will briefly review next.

**Fig. 1:**
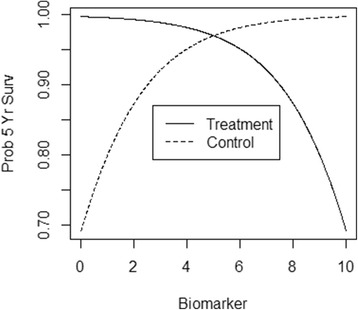
A predictive biomarker example. The solid line shows the probability of 5-year survival under treatment; as the biomarker value increases from 0 to 10, the probability of survival decreases. The dashed line shows the probability of 5-year survival under a control; as the biomarker value increases from 0 to 10, the probability of survival increases. The optimal therapy is: treatment those with biomarker value below 5, and do not treat those with biomarker value above 5

A predictive biomarker is clinically useful to the extent that there is a particular type of statistical interaction between the biomarker values and the effect of the treatment. Designing and analyzing studies to assess an interaction is more difficult than for more standard phase III clinical studies which test a main effect. But progress has recently been made [6–12]. For example, Fig. 1 shows the relationship between a hypothetical predictive biomarker’s values and the probability of 5-year survival, broken down by treatment groups. From this one can assess the variability in response to each treatment as a function of marker value. A similar figure appears in Janes et al. [10]. The optimal biomarker-guided treatment would be that individuals with marker value below 5 should receive treatment, and those with values above 5 should avoid the therapy. Since the improvement in 5-year survival based on optimal marker-guided therapy results from assigning those with marker value below 5 to the treatment, the distance between the “Control” and “Treatment” curves to the left of 5 characterizes the impact of the marker. Software for estimating this difference is available (TreatmentSelection, http://labs.fhcrc.org/janes/index.html).

### Type of data obtained/readout

This primer has focused on biomarkers that provide a continuous response since this is likely to be the most frequent scenario for immune-oncology biomarkers. These types of biomarkers include univariate “machine readout” settings, some pathologist scoring settings, multiplex assays and high dimensional assays (e.g., RNAseq). The latter two are included in this category because the multiple dimensions must be formed into a univariate score in order for clinical decisions to be made. This is typically done using a linear combination. In addition, a cutoff point to be used for the medical treatment decision must be specified. While the linear combination is typically formed based on statistical criteria, the selection of the cutoff point should be based largely on implications for clinical outcomes (e.g., lower tolerance for False Negative considering the life threatening nature of cancer and lack of alternative treatments for advanced tumors). Other types of biomarkers provide binary or categorical results.

The readout from software such as TreatmentSelection is the change in the average probability of 5-year survival under marker-guided therapy compared to current standard of care. The “average” here is taken across the target population.

### Limitations of the approach

**Fig. 2:**
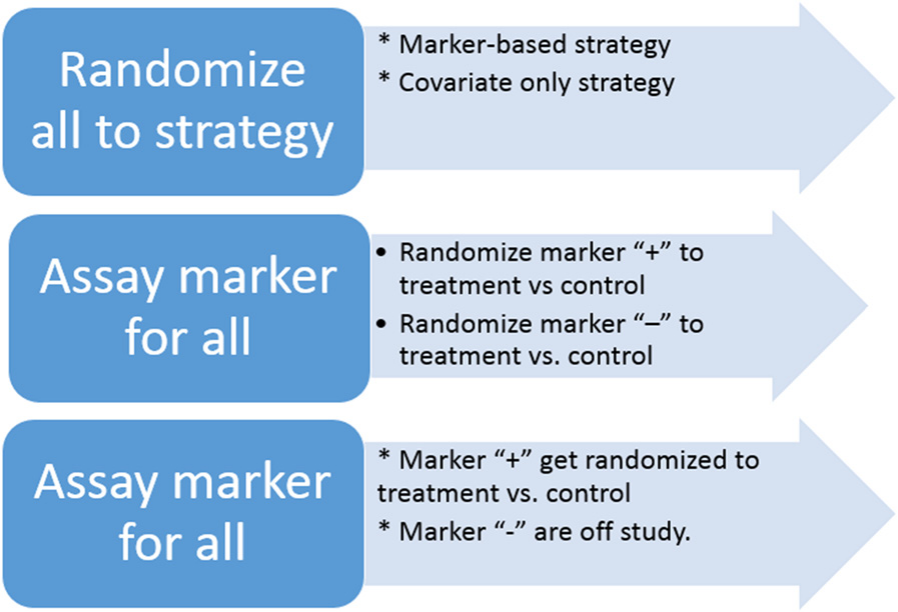
Different biomarker designs: Marker strategy design, marker stratified design, and enrichment design (top to bottom)

Biostatistical methods are not yet as well developed for predictive markers as they are for other types of markers. Common examples of biomarker study designs are shown in Fig. 2. The marker strategy design compares a standard of care strategy versus a marker guided strategy, and is being used in the MINDACT trial [13]. The marker stratified design provides the most complete information about the biomarker but tends to be the most expensive. The enrichment design uses the marker for patient selection.

### Types of samples needed and special issues pertaining to samples

In most cases, clinical validation of a predictive marker will require samples from a phase III clinical trial in which individual patients have been randomized to the therapy to which the marker is predicting response.

### Level of evidence

The number of publications discussing the methodologic aspects of the process of clinical validation of predictive biomarkers has been growing in parallel with the increasing understanding of the disease biology and the mechanism of action of cancer drugs, including immunotherapy approaches.

## References

[1] Dancey JE, Dobbin KK, Groshen S, Jessup JM, Hruszkewycz AH, Koehler M, Parchment R, Ratain MJ, Shankar LK, Stadler WM True LD, Gravell A, Grever MR on behalf of the Biomarkers Task Force of the NCI Investigational Drug Steering Committee (2010). Guidelines for the development and incorporation of biomarker studies in early clinical trials of novel agents. Clin Cancer Res.

[2] Raunig DL, McShane LM, Pennello G, Gatsonis C, Carson PL, Voyvodic JT, Wahl RL, Kurland BF, Schwarz AJ, Gonen M, Zahlmann G, Kondratovich M, O’Donnell K, Petrick N, Cole PE, Garra B, Sullivan DC and the QIBA Technical Performance Working Group (2014). Quantitative imaging biomarkers: A review of statistical methods for technical performance assessment. Stat Methods Med Res.

[3] McShane LM, Altman DG, Sauerbrei W, Taube SE, Gion M (2005). Clark GM for the Statistics Subcommittee of the NCI-EORTC Working Group on Cancer Diagnostics. J Natl Cancer Inst.

[4] Cook TD, DeMets DL (2008). Introduction to Statistical Methods for Clinical Trials.

[5] Simon RM, Paik S, Hayes DF (2009). Use of archived specimens in evaluation of prognostic and predictive biomarkers. J Natl Cancer Inst.

[6] Royston P, Sauerbrei W (2004). A new approach to modeling interactions between treatment and continuous covariates in clinical trials using fractional polynomials. Stat Med.

[7] Bonetti M, Gelber RD (2004). Patterns of treatment effects in subsets of patients in clinical trials. Biostatistics..

[8] Taylor JM, Ankerst DP (2008). Andridge RR Validation of biomarker-based risk prediction models. Clin Cancer Res..

[9] Song X, Pepe MS (2004). Evaluating markers for selecting a patient’s treatment. Biometrics..

[10] Janes H, Brown MD, Huang Y, Pepe MS (2014). An approach to evaluating and comparing biomarkers for patient treatment selection. Int J Biostat.

[11] Janes H, Pepe MS, Bossuyt PM, Barlow WE (2013). Measuring the performance of markers for guiding treatment decisions. Ann Intern Med.

[12] Janes H, Pepe MS, Huang Y (2014). A framework for evaluating markers used to select patient treatment. Med Decis Making.

[13] Cardoso F, Van’t Veer L, Rutgers E, Loi S, Mook S, Piccart-Gebhart MJ (2008). Clinical application of the 70-gene profile: the MINDACT trial. J Clin Oncol..

