# Erratum to: Immune monitoring technology primer

**DOI:** 10.1186/s40425-015-0100-2

**Published:** 2015-11-17

**Authors:** Kevin K. Dobbin

**Affiliations:** College of Public Health, University of Georgia, Athens, 30602, GA USA

The title of the original version of this article [1] contained an error. The correct title should be “Immune Monitoring Technology Primer: Clinical Validation for Predictive Markers.” The title has been corrected in the original article also.
